# An affordable and adaptable Faraday isolator design for research

**DOI:** 10.1016/j.ohx.2025.e00623

**Published:** 2025-01-24

**Authors:** Nicholas L. Wong, Ben Delaney, Takanori Miyazaki, Emma Sokell, Fergal O’Reilly

**Affiliations:** School of Physics, Science Centre North, University College Dublin, Belfield Dublin 4, Ireland

**Keywords:** Optical isolator, 3D printed, Faraday rotator, Stokes parameter, Mueller matrix

## Abstract

Presented here is an affordable Faraday isolator designed to account for conditions of high pulse energy and high total power lasers, with a clear input aperture allowing beam diameters up to 12 mm, and pulse energies and total powers limited by the dielectric crystal. This Faraday isolator is meant for laboratories with limited resources yet still need the features of commercial Faraday isolators. The design consists of a 3D printed mount housing seven permanent neodymium ring magnets, a Terbium Gallium Garnet (TGG) dielectric crystal, and two polarizing beam splitter cubes. Additionally, the design is customizable for different laser parameters, with the presented example isolator made for 1064 nm lasers. Measurements of the extinction ratio and a Stokes parameter analysis from different points within the isolator validated and characterized the constructed Faraday isolator. The final design had a measured minimum extinction ratio of 31.5±0.3 dB and a maximum of 39.9±0.2 dB depending on the polarization of back reflected light.

## Graphical abstract

**Figure:**
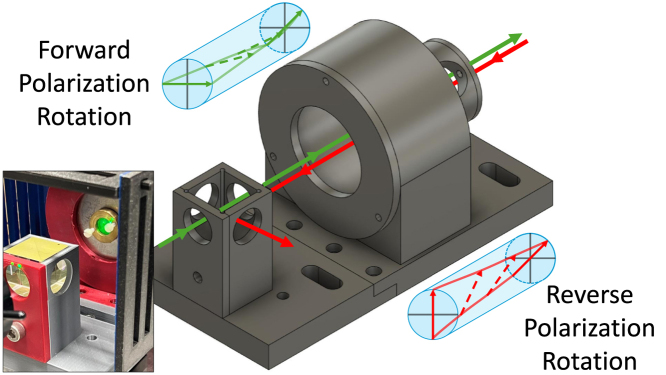

## Specifications table

**Table:** 

Hardware name	Faraday Isolator
Subject area	• Engineering and material science • Educational tools and open source alternatives to existing infrastructure

Hardware type	• Mechanical engineering and materials science

Closest commercial analog	Thorlabs IO-10-1064-VHP, Newport ISO-FRDY-08-1064-N, ConOptics 815 Optical Isolator

Open source license	CC-BY-4.0

Cost of hardware	2470 USD (2283 Euro)

Source file repository	https://doi.org/10.5281/zenodo.14525339

## 1 Hardware in context

High pulse energy and high power lasers are widespread in research with a variety of applications. For example, lasers ionize materials to produce a target or state of interest in studies of DNA damage [1], [2] and spectroscopic experiments of chemical reaction dynamics [3]. Lasers also probe targets in biological imaging [4], [5], laser-induced breakdown spectroscopy, [6], [7], and emission [8], [9] or absorption [10], [11] spectroscopy. Furthermore, lasers also have industry applications such as in extreme ultraviolet lithography in semiconductor production [12].

Many optical components are required for the application of lasers, and the component of interest here is the Faraday isolator. These devices are important in laser systems, which contain polarized light and have a chance for back reflections. In the case of high power or high pulse energy lasers, these back reflections can cause significant damage to optics or even the lasers themselves. Faraday isolators act as optical diodes preventing light from traveling in one direction, while allowing propagation in the other direction [13]. The cost of Faraday isolators can be prohibitive, with the price of isolators on the order of several 1000 s of euros. However, the design of a Faraday isolator is relatively simple. A Faraday isolator relies on the Faraday effect achieving a 45° angle of rotation of the polarization axis and two polarizing optics with transmission axes offset by 45°.

The Faraday effect is a rotation of the polarization angle of light passing through a dielectric material parallel to a magnetic field. The polarization angle of rotation, $\theta_{\mathrm{FR}}$ [rad], is defined as

(1) $$\theta_{\mathrm{FR}}=V\cdot B\cdot d,$$

where V is the Verdet constant — a material specific constant of the dielectric [rad/(T m)], B is the average magnetic field inside the dielectric material [T], and d is the length of the dielectric material [m]. The Verdet constant depends on the wavelength of the light; and importantly, the direction of polarization angle rotation is the same regardless of the direction of light propagation [13], [14]. Thus, the Faraday rotator consists of a magnetic field source and dielectric material within the magnetic field.

When two polarizers are placed on each side of the Faraday rotator, the two polarizers ensure light passing through the Faraday isolator is linearly polarized, and the consistent direction of the Faraday rotator’s angle of rotation ensures any light traveling in the reverse direction is orthogonally polarized to the input polarizer and consequently blocked. Therefore, the system forms an optical diode — the effect desired in high pulse and high power laser applications. An important aspect for practical applications is the imperfection of real polarizers and Faraday rotators, which leads to some amount of light passing through in the reverse direction; thus, proper construction of a 45° Faraday rotator is important to minimize this contribution.

Current research on these devices focuses on their application in specific scenarios. Many studies have focused on improving Faraday isolators for high average and peak power lasers. In this context, the Faraday isolators are being designed for laser powers on the order of several tens of W [15] to several kW [16], [17]. With such powers, a main issue for Faraday isolators is thermally induced effects such as Verdet constant changes, stress, and deformation, which can reduce the capabilities of the isolator [18], [19]. Thermally induced effects have been compensated for by adding additional optical elements to the Faraday isolator [19], [20]. Much research has also been conducted on different magneto-optical materials, since thermal effects are caused by the absorption of energy by the dielectric material. Two factors affect the severity of thermally induced effects, namely the absorption coefficient of the material and the length of the material [18]. On the one hand, the absorption coefficient has been addressed by the investigation of different materials [15], [16], [17], [18]. On the other hand, the length of the material has been addressed first by materials with high Verdet constants [18] and second by increasing the magnetic field strength allowing a shorter required material length [21]. The wavelength range of these studies can cover the visible range [16] but are mainly on the infrared range [18]. Thus, research to improve Faraday isolators primarily focuses on dielectric materials and compensation for thermally induced effects caused by high power lasers.

The hardware discussed here utilizes a Terbium Gallium Garnet (TGG) crystal as the dielectric material for a Faraday isolator designed for high power lasers up to 100 W at 1064 nm. So, thermal effects may need to be considered. A quantity known as $P_{max}$ gives the laser power where thermal effects cause the extinction ratio of a given material to reach a characteristic value, typically 30 dB; and $P_{max}$ is given as 700 W for a TGG crystal [18]. Therefore, with the given design constraints, thermally induced changes in the Verdet constant and depolarization caused by thermal stress and deformation will not need to be considered. However, the $P_{max}$ gives an indication of the upper limit of the Faraday isolator design.

Presented here is the design and characterization of an affordable and adaptable Faraday isolator. Advances in 3D printing technology facilitate the affordability aspect, by allowing the Faraday isolator housing to be designed using CAD software and then cheaply printed. In this design, the dielectric crystal is offset from the magnets at a specific axial distance to achieve the required magnetic field for $\theta_{\mathrm{FR}}=45{}^{\circ}$. The employment of an axial offset gives design freedom in the number and size of ring magnets, enabling the preferred dielectric medium to be employed and facilitates the adaptability of the Faraday isolator to different laser parameters. Thus, this new Faraday isolator design is intended to benefit research laboratories with limited resources but require one or more Faraday isolators with the capabilities of a commercial isolator.

**Fig. 1:**
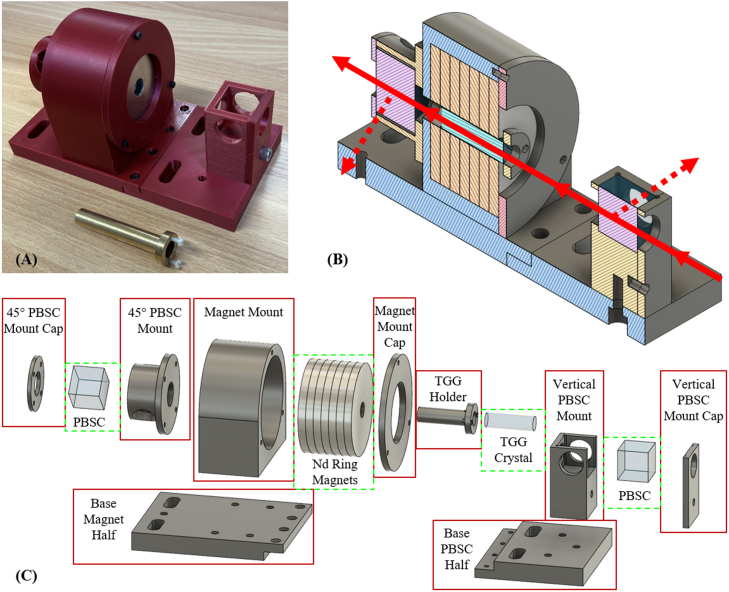
A picture of the 3D printed Faraday isolator with the seven Nd ring magnets placed inside the mount and the TGG crystal within a brass TGG Holder placed beside the isolator (A), a section view of the CAD design of the Faraday isolator with the intended normal operating direction or laser transmission direction (red arrow) and reflected light outputs (red dashed arrows) shown (B), and an exploded view of the CAD design (C). Section 5.2 contains more information on the intended operating direction shown in (B). In (C) 3D printed (or possibly machined in the case of the TGG holder, see Section 2) parts are labeled according to Section 3 and boxed in red, and purchased components are labeled according to Section 4 and dash boxed in green.

## 2 Hardware description

The presented Faraday isolator design:

• is an affordable alternative to commercially available Faraday isolators and optical diodes,
• provides a controllable and adjustable Faraday rotation setup,
• can be customized for different laser wavelengths and Faraday isolator components.

Specifically, the Faraday isolator presented here was constructed for an operating laser wavelength of 1064 nm. A 40 mm long, 12 mm diameter, Terbium Gallium Garnet (TGG) crystal from Northrop Grumman was the chosen dielectric material. The TGG crystal was specified by the supplier to have a V=−40 Rad/(T m) for 1064 nm light [22]. A stack of seven axially magnetized neodymium ring magnets (sintered Nd-Fe-B, grade N48) from Apex Magnets provided the magnetic field. The dimensions of the magnets were 3” outer diameter, 1/2” inner diameter, and 1/4” thickness (76.2 mm, 12.7 mm, and 6.35 mm respectively). The TGG crystal was placed within a brass mount called the TGG Holder, such that the dielectric was centered within the hole of the ring magnets. Further, the brass TGG Holder had two plastic screws to control the axial position of the TGG crystal, which was set such that the face of the TGG crystal outside the magnet stack was 3.0 mm from the face of the magnet stack. The magnets and brass TGG Holder with the TGG crystal can be seen in the assembled Faraday isolator at the bottom of Fig. 1(A). The TGG Holder can also be printed 3D printed, but the precision needs to be greater than 0.7 mm. Recently, TGG Holders were successfully printed using an Original Prusa SL1S SPEED resin printer with an Original Prusa CW1S Curing and Washing Machine, though the following experiments were performed with the brass holder.

For the housing and mounting of the Faraday rotator with respect to the polarizers, the type of polarizer employed determines any specific design stipulations. Two high energy polarizing beamsplitter cubes (PBSCs) filled the role of the input and output polarizers for the present Faraday isolator design. The PBSCs are from EKSMA Optics (model: 435–1223), are 25.4 mm on each side, have an operating wavelength of 1064 nm, and a transmission of P polarized and S polarized light of 99.7% and 0.5% at 1064 nm, respectively [23] A frame to hold the stack of ring magnets and two PBSCs was designed in AutoCAD Fusion and 3D printed on an Original Prusa i3 MK3 filament printer. The design geometrically offsets the optical axis of the PBSCs by 45°. Fig. 1(A) shows the final constructed Faraday isolator, Fig. 1(B) displays a section view of the Fusion design, and Fig. 1(C) contains an exploded view of the design with each part labeled according to Section 3.

***Design files***

## 3 Design files summary

**Table:** 

Design filename	File type	Open source license	Location of the file
Base Magnet Half	CAD and STL file	CC BY 4.0	https://doi.org/10.5281/zenodo.14525339
Base PBSC Half	CAD and STL file	CC BY 4.0	https://doi.org/10.5281/zenodo.14525339
Magnet Mount	CAD and STL file	CC BY 4.0	https://doi.org/10.5281/zenodo.14525339
Magnet Mount Cap	CAD and STL file	CC BY 4.0	https://doi.org/10.5281/zenodo.14525339
Vertical PBSC Mount	CAD and STL file	CC BY 4.0	https://doi.org/10.5281/zenodo.14525339
Vertical PBSC Mount Cap	CAD and STL file	CC BY 4.0	https://doi.org/10.5281/zenodo.14525339
$45^{\circ }$ PBSC Mount	CAD and STL file	CC BY 4.0	https://doi.org/10.5281/zenodo.14525339
$45^{\circ }$ PBSC Mount Cap	CAD and STL file	CC BY 4.0	https://doi.org/10.5281/zenodo.14525339
TGG Holder	CAD and STL file	CC BY 4.0	https://doi.org/10.5281/zenodo.14525339

The CAD files listed above are provided as STEP and AutoDesk Fusion files (F3Z and F3D). The first eight CAD and STL files compromise the 3D printed Faraday isolator housing, which can be seen in Fig. 1(C). The base of the housing is split into two halves for 3D printing ease and usefulness in some experimental and assembly scenarios. Base Magnet Half is the half of the base which the Magnet Mount attaches, and Base PBSC Half is the remaining half which connects to the Vertical PBSC Mount. Magnet Mount holds the seven ring magnets, and Vertical PBSC Mount and 45° PBSC Mount hold a PBSC each. Magnet Mount Cap, Vertical PBSC Mount Cap, and 45° PBSC Mount Cap all connect to their respective mounts to securely hold the magnets or PBSCs within the mounts. More detailed information on how the housing connects is in Section 5.1. The TGG Holder is either a brass holder or a 3D printed holder, which the TGG crystal is placed within. The provided CAD and STL files are for the 3D printed version, but the brass version would be similarly machined. The TGG Holder allows the axial positioning of the TGG crystal and centers the TGG crystal within the ring magnets. The brass holder has a slightly crimped edge to stop the TGG crystal from sliding, while the 3D printed holder has four small radial steps as part of the design.

***Bill of materials***

## 4 Bill of materials summary

**Table:** 

Designator	Component	Number	Cost per unit - currency	Total cost - currency	Source of materials	Material type
Nd Ring Magnet	SKU#: M31214R, $3^{\prime \prime }\times 1/2^{\prime \prime }\times 1/4^{\prime \prime }$, N48, Axially magnetized neoydmium ring magnet	7	34.91 USD	244.37 USD	Apex Magnets	Metal

TGG Crystal	40 mm long, 12 mm diameter, terbium gallium garnet dielectric crystal	1	1250 USD	1250 USD	Northrop Grumman	Dielectric

PBSC	435-1223	2	450 Euro	900 Euro	EKSMA Optics	Non-specific

The cost of the components are presented in the currency which their price is quoted in. While the components described in the table are the majority of the cost, several smaller items are required, namely fifteen M3 and five M6 screws and threaded inserts. While plastic screws would make assembly easier, metal screws are acceptable. An additional smaller cost is the PLA filament for the Faraday isolator 3D print, with the total weight used about 300 g (100 m), and the resin for the TGG Holder 3D print, with the total weight used about 4 g (4 ml) of resin.

**Fig. 2:**
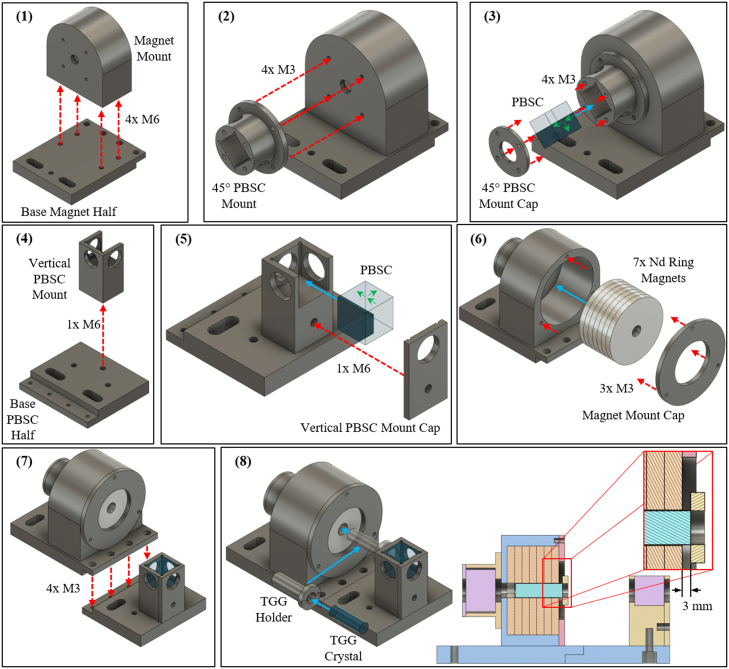
The recommended assembly procedure described in Section 5.1 is shown, with each numbered panel depicting the same numbered step in Section 5.1. The red dashed arrows indicate two parts being connected with screws, where the arrow head points to the threaded component. The blue arrows depict places where components should be inserted into another component. The green arrows on the PBSCs in steps (3) and (5) indicate the light transmission and reflection directions oriented with safety in mind as discussed in Section 5.2. Step (8) also contains a section view of the assembled Faraday isolator along with an inset indicating the 3 mm offset position of the TGG crystal edge with respect to the magnet’s edge as described in Section 5.1 step 8(a).

## 5 Build instructions

### 5.1 Assembly

Assembly of the Faraday isolator begins with 3D printing. The eight CAD design files not including the TGG Holder described in Section 3 should be converted to STL files and printed on the 3D printer of choice. The Faraday isolator presented here was printed on a Prusa i3 MK3, which necessitated the conversion of the STL files to gcode files. With the 3D printed parts, the M3 and M6 threaded inserts should be placed into the respective components: four M3 inserts in Base PBSC Half, three M3 and four M6 inserts in Magnet Mount, two M6 inserts in Vertical PBSC Mount, and at least six M3 inserts in 45° PBSC Mount (two screws can hold the 45° PBSC Mount Cap). To place the inserts, a soldering iron with a diameter approximately the size of the insert hole (three or six mm) should be inserted into the hole to be threaded first for a few seconds to partially heat the plastic. Then, after removing the soldering iron, the insert should be pressed into the hole. One last step before assembly the Faraday isolator is to create the magnet stack. For the Nd ring magnets utilized here, the strength of the magnetic field posed a safety hazard if fingers were caught between magnets coming together, and the acceleration of two magnets coming together could also shatter one of the ring magnets. Thus, when making the magnet stack, the magnets should be slowly brought together, for example with a guide and wooden or plastic wedges. Importantly, the strength of these magnets should be kept in mind when assembly or working near the Faraday isolator, as metal objects and tools can easily be pulled to the magnet stack.

**Fig. 3:**
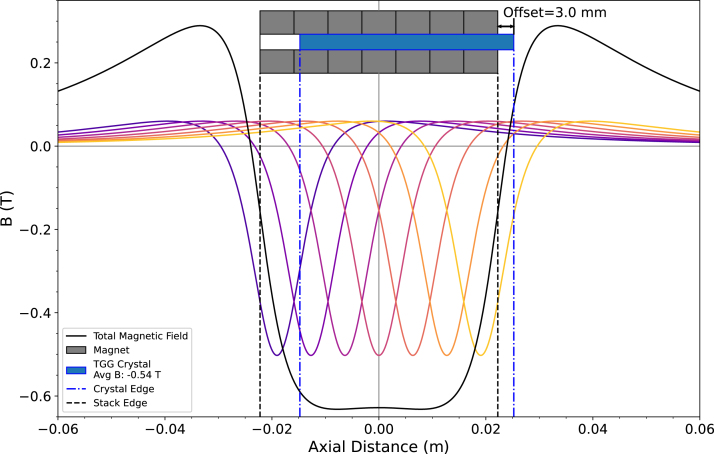
The magnetic field of the total stack of seven magnets (black solid curve) and the individual magnets of the stack (colored solid curves). Vertical lines denote the axial edges of the magnet stack (black dashed line) and the edges of the TGG crystal (blue dash-dotted line). Additionally, a depiction of the magnet stack is shown at the top of the plot, with the TGG crystal’s 3.0 mm offset labeled. The magnets (gray rectangles) and TGG crystal (blue rectangle) images are not to scale in vertical dimensions, but are axially to scale. The average magnetic field experienced by the TGG crystal is −0.54 T. (For interpretation of the references to color in this figure legend, the reader is referred to the web version of this article.)

The components can then be connected. While the order of assembly is not rigidly fixed, the following order, which is also depicted in Fig. 2, may make the process easier:

1. The Magnet Mount should be attached to the Base Magnet Half, with four M6 screws.
2. The 45° PBSC Mount should be attached to the Magnet mount with at least two M3 screws.
3. One of the PBSCs should be placed into the 45° PBSC Mount and secured by screwing in the 45° PBSC Mount Cap with at least two M3 screws.
  (a) When placing this PBSC, ensure the reflected transmission direction is through one of the open ports on the 45° PBSC Mount, not one of the blocked directions, and that the primary transmission direction is through the axis of the Magnet Mount.
4. The Vertical PBSC Mount should be attached to the Base PBSC Half with a single M6 screw.
5. The remaining PBSC should be placed into the Vertical PBSC Mount and secured with the Vertical PBSC Mount Cap.
  (a) Again, the primary transmission direction of the PBSC should be through the axis of the Magnet Mount.
6. The stack of magnets should be placed into the Magnet Mount and secured with the Magnet Mount Cap and three M3 screws.
  (a) The direction of the magnetic field with respect to the orientation of the PBSCs determines which direction the transmission direction of the Faraday isolator, and a simple experiment, described in Section 5.2, can determine the transmission direction.
  (b) Ideally, the transmission direction should go from the Vertical PBSC side to the 45° PBSC side as shown in Fig. 1(B).
  (c) While the Base PBSC Half can be connected to the Base Magnet Half after the magnets have been placed, the transmission direction check experiment can be more straightforward with the two halves still separate.
7. Once the magnet stack is in the desired orientation, the Base PBSC Half and Base Magnet Half can be joined with four M3 screws.
8. The TGG crystal can be placed into the TGG Holder and both placed within the magnet stack on the magnet side opposite the 45° PBSC.
  (a) The screws should be adjusted such that the face of the TGG crystal outside the magnet stack is 3.0 mm away from the face of the closest magnet. Fig. 2 step (8) and Fig. 3 depict the offset distance in the upper part of the plot.
  (b) The distance of 3.0 mm was determined through magnetic field modeling and experimentally, which are described in Sections 5.3, 7, respectively.

### 5.2 Direction check

The forward operating direction, or transmission direction, of the Faraday isolator is the laser propagation direction through the Faraday isolator which is designed for maximum transmission. Meanwhile, the opposite propagation direction is designed for maximum PBSC reflection. The transmission direction should be from the Vertical PBSC side to the 45° PBSC side, as indicated by Fig. 1(B), to help with safety. The first PBSC which the laser pulse encounters is the most likely to redirect more significant fractions of the initial laser intensity; thus, by having the first PBSC be the Vertical PBSC, the reflected light path is along traditional beam paths — namely parallel to the laboratory table. This facilitates the easy deployment of an appropriate beam dump.

The transmission and reflection directions are determined by the magnetic field direction, or in the present case the orientation of the magnet stack. The transmission direction can be determined simply with a 1064 nm laser and an energy detector or photodiode. The fully assembled Faraday isolator should be placed in front of the laser and oriented such that the Vertical PBSC Mount is the first PBSC encountered in the beamline. The detector is placed on the other side of the isolator. Then, the laser should be fired and the energy recorded. Next, the Faraday isolator should be rotated 180°, making the 45° PBSC Mount now first, and, the laser should be fired and energy recorded again. Whichever orientation yielded the highest energy is the transmission direction of the Faraday isolator. If the operating direction is opposite of the desired direction, then the magnet stack needs to be removed, flipped 180°, and replaced into the Magnet Mount. If the magnetic field is measured, then it should now be the opposite sign, and repeating the direction check experiment should yield the other desired result.

### 5.3 Customization

The Faraday isolator design presented here can be customized for different lasers. The operation of a Faraday isolator is primarily dependent on the design of the Faraday rotator and the housing or mounting of the Faraday rotator with respect to the two polarizers. Utilizing a housing design, such as the one described in Section 2 provides an adaptable mount, with the polarizing beam splitters’ axes physically offset by 45° and the required magnetic field can be adjusted by the position of the dielectric crystal. Thus, the Faraday rotator can be adjusted to customize the Faraday isolator, with minimal changes to the design. For example, the Vertical PBSC Mount could be directly connected to the Magnet Mount Cap to allow for easier rotation of the Faraday isolator.

Eq. (1) underscores the two primary aspects of a Faraday rotator which enable easy customization. The dielectric material gives the Verdet constant and the length, V and d respectively; and the magnetic field source gives the magnetic field throughout the dielectric, B. Thus, by choosing a dielectric crystal to use, V and d are constant, and Eq. (1) produces an exact magnetic field that causes a 45° rotation of the polarization vector. By placing the dielectric partially inside and partially outside the magnets, the magnetic field determined from Eq. (1) can be more easily achieved with a wider range of dielectric lengths. However, modeling the magnetic field of the ring magnets is essential due to the specificity of the required magnetic field. For the Faraday isolator design presented here, axial magnetic field models from Winter, Mok, and Kumarakrishnan [13] and Babic and Akyel [24] aided in the design process, by giving an expected axial position of the TGG crystal for 45° rotation. The residual magnetic field, or remanence, is the maximum magnetic field of a permanent magnet in the absence of other magnetic fields [25], [26] and is required for both magnetic field models either to calculate the magnetic moment [13] or as an approximation of the surface magnetic pole density [24], [27]. A remanence of 13.7 kG was employed in the magnetic field modeling based on the reported range of sintered Grade N48 Nd-Fe-B magnets of 13.6 to 14.3 kG [28], [29] and good agreement with direct magnetic field measurements. The axial magnetic field model of Babic and Akyel also requires a non-zero radial distance [24], so a very small radial distance of $1\times 10^{-6}$ m was used. The results of the Babic and Akyelmodel [24] are shown in Fig. 3. The magnetic field curves in Fig. 3 highlight the stack’s total magnetic field having large positive and negative areas with a sharp slope between them, which enables the dielectric axial offset method to vary the average magnetic field experienced by the dielectric material.

An example customization design process thus starts by determining what dielectric crystal is needed based on the laser parameters and choosing a length of the dielectric. Then, Eq. (1) yields the required magnetic field for the chosen length of the dielectric. Next, the magnetic field of ring magnets which fit the dielectric within their center should be modeled, for example with the models from [13], [24]. In the magnetic field modeling, the average magnetic field over the length of the dielectric should be calculated at different axial positions of the dielectric crystal to determine the required dielectric position and number of magnets. These first steps can be iterated to determine the optimal dielectric crystal length and type of magnets for experimental constraints and budgets. Finally, with the chosen number of magnets, the Magnet Mount 3D design file should be adjusted to accommodate the number and size of magnets. The new design should maintain the axial centering of the magnets with respect to the PBSCs’ transmission axis. The Faraday isolator can then be assembled as described previously.

Importantly, the magnetic field models utilized here assume the dielectric crystal is axially centered within the ring magnets. So, when choosing new magnets and dielectrics, either the dielectric should have a snug fit, a centering holder should be made, or the magnetic field model should account for the change in position. For the work presented here, a brass holder was created as described in Section 2. However, the magnetic field modeling did not account for the TGG Holder, and experimental results directly measuring the magnetic field showed a noticeable effect on the magnetic field experienced by the TGG crystal. For the customization process, instead of performing more intensive modeling of the magnetic field, a simple extinction ratio experiment shows the optimum dielectric crystal position. The experiment is described in more detail in Section 7.2, since the extinction ratio test not only reveals the needed dielectric position but also characterizes the Faraday isolator’s extinction ratio — a common figure of merit. Thus, the custom Faraday isolator could also be primarily designed through experimental verification after initial magnetic field modeling.

## 6 Operation instructions

After assembly and once the transmission direction of the Faraday isolator has been determined as in Section 5.2, operation of the Faraday isolator can be as simple as placing the isolator within a beamline with the transmission direction aligned with the forward laser propagation direction. However, several additional components can achieve more ideal operational conditions.

The first additional component is half-waveplate. The half-waveplate should be placed before the Faraday isolator in the beamline. A mount for the half-waveplate could even be added to the 3D printed Faraday isolator model. The half-waveplate is used to adjust the linear polarization angle of the laser relative to the transmission axis of the PBSC within the Vertical PBSC mount (Vertical PBSC) to allow for variable throughput. The second important component is for safety. Even if a half-waveplate is employed as stated previously, a beam dump should be placed in the direction of the reflected beam of the Vertical PBSC in the Faraday isolator, since large amounts of the laser’s energy could be redirected here. The beam dump should be appropriately chosen for the laser in use, accounting for the laser pulse energy, duration, size, and frequency. Lastly, the orientation of the PBSC in the 45° PBSC Mount (45° PBSC) should be considered. Light reflected at the 45° PBSC will propagate either into the optical bench or towards the ceiling at 45° posing a safety hazard. The orientation of the 45° PBSC determines whether light traveling in the transmission direction or light traveling in the reverse direction is reflected towards the ceiling. In either case, reflection paths should be oriented away from sensitive equipment or common areas for personnel. Placing a beam dump in both reflection paths ensures maximum safety. It is not recommended to use 3D printed parts to block the ports of the 45° PBSC Mount, because lasers can easily melt the plastic and damage the PBSC.

An additional precautionary component can be 3D printed. A simple cage can be made to surround the entire Faraday isolator, which helps prevent tools from being brought to close to the strong magnets of the Faraday isolator. This component is not necessary, but can make experimental work around the Faraday isolator easier.

## 7 Validation and characterization

### 7.1 Stokes parameter validation

**Fig. 4:**
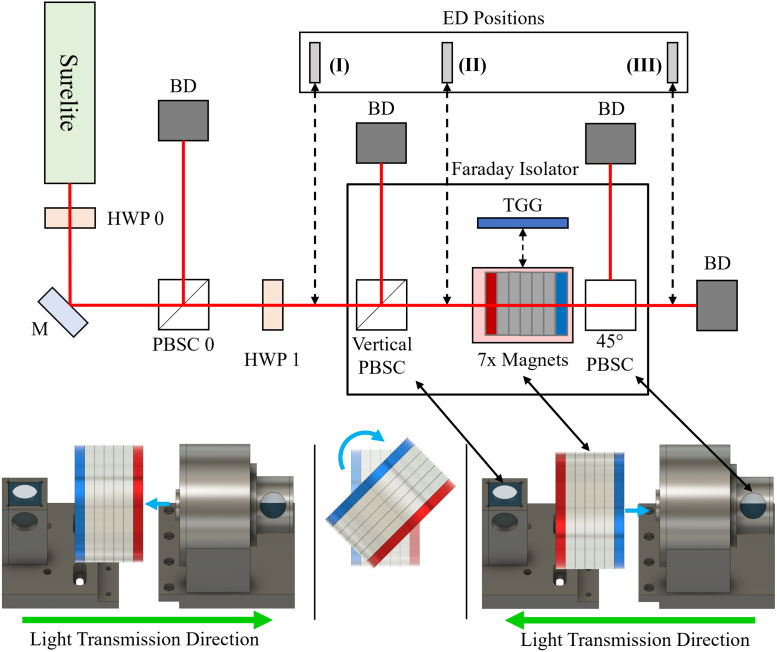
Optical schematic for the Stokes parameter and extinction ratio tests (upper half), and a CAD design depiction of the Faraday isolator set up for the tests (lower half). Surelite is the Surelite 1064 nm laser, M is a mirror, HWP is a half-waveplate, PBSC is a polarizing beam splitter cube, BD is a beam dump, TGG is the TGG crystal, 7x Magnets denotes the magnets of the Faraday rotator with or without the TGG crystal placed inside as indicated by the dashed arrow, and ED Positions marks the positions the energy detector (ED) was placed. 45° PBSC is the PBSC whose optical axis is rotated 45° with respect to Vertical PBSC. The CAD design images in the lower part of the figure show the removal of the magnet stack, rotation of the magnets, and replacement of the stack, where the blue arrows indicate the movement of parts, the black arrows point to analogous components in the optical schematic, and the green arrows are the isolator’s light transmission direction. The rotation of the magnet stack is done for the laser and personnel safety reasons described in Sections 7.1, 5.2. In the CAD design and schematic, the two end magnets are colored blue and red. The colors do not denote a specific direction of the magnetic field and are only meant to highlight the rotation of the magnet stack.

The important aspect of the Faraday isolator is its capabilities as an optical diode. So, validation of the Faraday isolator consists of testing how well the constructed Faraday isolator handles back reflected light. Utilizing a beampath that reflects light back through the Faraday isolator could damage the laser employed in the tests. Instead, for the safety of the laser, the Faraday isolator was reversed, such that the laser propagation direction was anti-parallel to the transmission direction of the Faraday isolator. The magnet stack of the Faraday isolator was rotated 180° to achieve the reversed direction and maintain experimental safety. Experimental tests utilized the optical schematic shown in Fig. 4, and the lower half of Fig. 4 displays the rotation of the magnet stack to change the transmission direction of the isolator. A Surelite laser provided a 1064 nm laser pulse, which passed through an initial half-waveplate (HWP 0) and polarizing beam splitter cube (PBSC 0) to decrease the energy of the laser pulse and ensure the laser light is linearly polarized. The laser pulse then passed through another half-waveplate (HWP 1) followed by the Faraday isolator, containing the Vertical PBSC, 45° PBSC, and Faraday rotator consisting of the 7 magnets and TGG crystal.

The laser beam energy was recorded with a Gentec-eo QE25SP-S-MB energy monitor at three different locations: before the Faraday isolator but after HWP 1, after the output PBSC (Vertical PBSC) of the Faraday isolator, and after the Faraday isolator, positions **(I)**, **(II)**, and **(III)**, respectively, shown in Fig. 4. Three laser shots were measured at each position. Measurements at **(III)** were recorded both when the TGG crystal was in the magnets and the when the TGG crystal was removed. When the energy monitor needed to be placed after the Vertical PBSC, the Vertical PBSC was placed separately from the Faraday isolator, but in the same orientation that the PBSC would be in within the isolator. The removal of the Vertical PBSC is pictured in the CAD design insert of Fig. 4 as the separation of the two halves of the Faraday isolator. HWP 1 was rotated to maximize or minimize the energy transmission through the Vertical PBSC measured at **(II)**, corresponding to P (0° with respect to horizontal) or S (90°) polarized light, respectively.

Experimental results from the three different positions were compared to expected values from a simulated Faraday isolator. The simulation was performed using Stokes parameters, Mueller matrices, and Mueller calculus. The Mueller formalism was chosen over the Jones formalism, because generally Jones calculus is better suited for optical systems where the sum of electric field amplitudes is the focus while Mueller calculus is more suited for the sum of intensities [30], which is the case here. Stokes parameters describes the polarization state of a laser pulse with four parameters, which can be represented as a vector [30]. The four Stokes parameters are $S_{0}=$ the total intensity, $S_{1}=$ the amount of horizontally or vertically polarized light, $S_{2}=$ the amount of +45° or −45° polarized light, and $S_{3}=$ the amount of right- or left-circularly polarized light [30]. Commonly, the Stokes parameters are normalized to $S_{0}$, making the range of Stokes parameters $0\leq S_{0}\leq 1$ and $-1\leq S_{1},S_{2},S_{3}\leq +1$ [30]. Each optical component in a beamline is represented as a 4 × 4 matrix known as a Mueller matrix [30]. Mueller calculus involves performing matrix multiplication on a Stokes vector, which describes how the optical component changes the polarization of light described by the Stokes vector [30].

Since only the total intensity was recorded at different positions, normalizing the results of **(II)** or **(III)** to the energy recorded at **(I)** yields experimental $S_{0}$ values normalized to **(I)**. The experimental ratios were then compared to $S_{0}$ at the same positions of a simulated Faraday isolator, normalized and starting at **(I)**. Several conditions needed to be set before the comparison could be done, namely the initial Stokes parameters and the Mueller matrices describing the optical components of the Faraday isolator, specifically the Faraday rotator and the two PBSCs.

The Stokes parameters were assumed to be fully linearly polarized due to the initial optics (HWP 0 and PBSC 0) and fully P or S polarized due to the experimental maximization or minimization with HWP 1 and the Vertical PBSC. Thus, the initial Stokes vector was either [1,1,0,0] or [1,–1,0,0] corresponding to P or S polarized light. The Faraday rotator of the Faraday isolator was represented by the Mueller matrix:

(2) $$M_{FR}=\left[\begin{array}{cccc} 1 & 0 & 0 & 0\\ 0 & \cos\left(2\theta_{\mathrm{FR}}\right) & -\sin\left(2\theta_{\mathrm{FR}}\right) & 0\\ 0 & \sin\left(2\theta_{\mathrm{FR}}\right) & \cos\left(2\theta_{\mathrm{FR}}\right) & 0\\ 0 & 0 & 0 & 1 \end{array}\right],$$

where $\theta_{\mathrm{FR}}$ is the same polarization angle of rotation due to the Faraday effect described in Eq. (1) [30], [31], in units of degrees here [${}^{\circ }$]. $\theta_{\mathrm{FR}}$ was set to 45° for the simulated Faraday isolator, representing a perfect Faraday rotation. While the Vertical PBSC and 45° PBSC can be described by Mueller matrices derived from minimum expected performance values reported by EKSMA optics, utilizing experimental determined parameters yields a more accurate simulation. Thus, the two PBSCs were represented by non-ideal general linear polarizer Mueller matrices, which can utilize either reported or expected performance values for comparison:

(3) $$M_{P}\left(\theta \right)=\left[\begin{array}{cccc} A & B\cos\left(2\theta \right) & B\sin\left(2\theta \right) & 0\\ B\cos\left(2\theta \right) & A\cos^{2}\left(2\theta \right)+C\sin^{2}\left(2\theta \right) & \left(A-C\right)\cos\left(2\theta \right)\sin\left(2\theta \right) & 0\\ B\sin\left(2\theta \right) & \left(A-C\right)\cos\left(2\theta \right)\sin\left(2\theta \right) & A\sin^{2}\left(2\theta \right)+C\cos^{2}\left(2\theta \right) & 0\\ 0 & 0 & 0 & C \end{array}\right],$$

here θ [${}^{\circ }$] is the angle of rotation of the linear polarizer or the polarizer’s transmission axis and A, B, and C [unitless] are coefficients which can be related to the polarizer’s principal transmittance parameters $k_{1}$ and $k_{2}$ [30], [32] by

(4) $$A=\frac{k_{1}+k_{2}}{2},$$

(5) $$B=\frac{k_{1}-k_{2}}{2},$$

(6) $$C=\sqrt{k_{1}k_{2}}.$$

$k_{1}$ and $k_{2}$ [unitless] can be determined experimentally, by directing a light source through a half-waveplate before the polarizer and measuring the maximum and minimum intensity after the polarizer as the half-waveplate is rotated [30]. Thus, the transmittance parameters are

(7) $$k_{1}=\left[A+B\right]=\frac{I_{max}}{I_{0}},$$

(8) $$k_{2}=\left[A-B\right]=\frac{I_{min}}{I_{0}},$$

and in these relationships $k_{1}$ and $k_{2}$ [unitless] are the major and minor transmittance parameters of the linear polarizer, $I_{0}$ is the initial intensity of the light source [W/cm^2^], and $I_{max}$ and $I_{min}$ [W/cm^2^] are the maximum and minimum intensity recorded after the polarizer [30]. The ratio of the transmittance parameters are often given as specifications of linear polarizers, where $R_{t}=k_{1}/k_{2}$ is the principle transmittance ratio and the inverse ($1/R_{t}$) is the extinction ratio. Importantly, this is not the same Faraday rotator extinction ratio discussed in Section 7.2. EKSMA Optics report an extinction ratio of at worst 1/500, analogous to $R_{t}>500/1$ [23]. Since $k_{1},k_{2}\leq 1$ according to Eqs. (7), (8), the minimum performance transmittance parameters for the PBSCs are $k_{1}=1$ and $k_{2}=1/500$. Eqs. (4)–(6) yield coefficients of A=0.5010, B=0.4990, and C=0.04472 from the minimum performance transmittance parameters.

The last Mueller matrix required for the simulation accounts for the anti-reflection coating on the surfaces of the PBSCs and TGG crystal. Both EKSMA Optics and Northrop Grumman report their optical components as having anti-reflection coatings with less than 0.25% reflections, corresponding to transmissions greater than 399/400 [22], [23]. The Mueller matrix which accounts for a loss of transmission is simply an identity matrix with a coefficient

(9) $$M_{P}\left(\theta \right)=T\left[\begin{array}{cccc} 1 & 0 & 0 & 0\\ 0 & 1 & 0 & 0\\ 0 & 0 & 1 & 0\\ 0 & 0 & 0 & 1 \end{array}\right],$$

where T [unitless] is the transmission rate [30]. So, in the Mueller calculus of the Faraday isolator, anti-reflection coatings can be represented solely as scalar coefficients, T=399/400. Since the laser pulse passes through two surfaces at each PBSC and TGG crystal, the coefficient will be applied twice for each component.

With these Mueller matrices, the Faraday isolator can be fully simulated. θ was set to 0° for the Vertical PBSC and −45° for 45° PBSC to yield a positive Faraday rotation in Eq. (3). Then, the output of the full simulated Faraday isolator is calculated as

(10) $$S^{III}=T^{6}M_{P}\left(\theta =-45{}^{\circ}\right)M_{FR}\left(\theta_{\mathrm{FR}}=45{}^{\circ}\right)M_{P}\left(\theta =0{}^{\circ}\right)S,$$

where **S**${}^{III}$ [unitless] is the Stokes vector after passing through the Faraday isolator at position **(III)** and **S** [unitless] is the initial Stokes vector at position **(I)**. When the isolator was simulated for the case without the TGG crystal, $M_{FR}$ and two Ts were omitted from the simulated isolator. For **(II)**, only the first Mueller matrix and two Ts are required, that is

(11) $$S^{II}=T^{2}M_{P}\left(\theta =0{}^{\circ}\right)S,$$

and here **S**${}^{II}$ [unitless] is the Stokes vector after passing through the Faraday isolator at position **(II)**. As stated previously, only the intensity was recorded at each position, and of the Stokes parameters only $S_{0}$ is compared between experiment and simulation. For these comparisons, the superscript will denote the position in the beamline the $S_{0}$ value is taken at, that is $S_{0}^{II}$ for position **(II)** and $S_{0}^{III}$ for **(III)**.

**Fig. 5:**
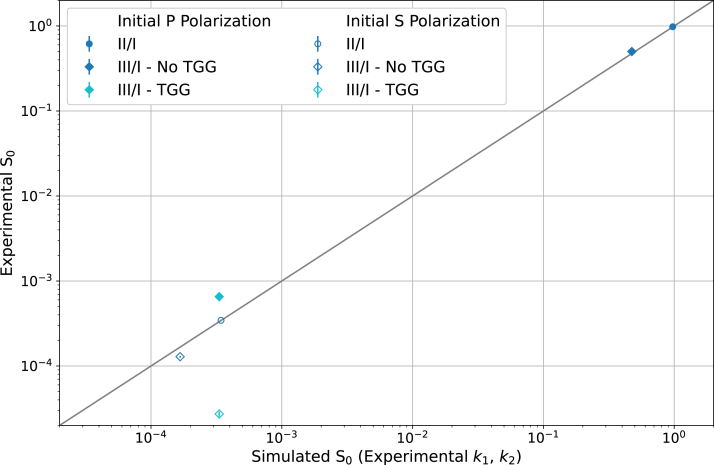
$S_{0}$ comparison between experimentally recorded values and simulated values with experimentally measured transmittance parameters. Experimental $S_{0}$ values come from ratios of energies at different positions, namely **(II)**/**(I)**$=S_{0}^{II}$ (circles) and **(III)**/**(I)**$=S_{0}^{III}$ (diamonds). The initial polarization of the light was either P (filled shapes) or S (empty shapes) polarized. Additionally, for $S_{0}^{III}$ the measurements were made either without the TGG crystal in place (blue) or with the TGG crystal (cyan); the $S_{0}^{II}$ cases are blue, since the TGG crystal has no influence on the results. The gray line indicates the ideal case of experiment and simulation matching one-to-one. (For interpretation of the references to color in this figure legend, the reader is referred to the web version of this article.)

With both experimental and simulated results defined, values for both the results are presented in Table 1. Eqs. (7), (8) show that the experimental ratio of **(II)**/**(I)** yields a measure of the EKSMA Optics PBSCs’ major and minor transmittance parameters. The experimental ratios directly give parameters of $k_{1}=0.98\pm 0.02$ and $k_{2}=3.45\times 10^{-4}\pm 0.06\times 10^{-4}$, as shown in Table 1. For comparison, the reported minimum performance principle transmittance of the PBSCs is $R_{t}=500$ [23], and the experimental results yield $R_{t}=2840\pm 70$. Thus, the PBSCs perform better than their reported minimum performance, as expected. Given the better accuracy, the following analysis focuses on the results with the experimental $k_{1}$ and $k_{2}$. The Faraday isolator was simulated with the experimentally determined $k_{1}$ and $k_{2}$, and the values are displayed Table 1. A comparison of simulated and experimental $S_{0}$ is shown in Fig. 5

First, Table 1 highlights the greater agreement between the experimental and simulated results, when the experimentally determined $k_{1}$ and $k_{2}$ are employed. Specifically, the experimental transmittance parameters bring the smaller value simulated $S_{0}$ values closer to the experimental results.

**Table 1:** Experimentally measured and simulated $S_{0}$ values for different Faraday isolator positions and conditions. Simulated values were calculated using minimum performance parameters from EKSMA Optics (Reported Transmittance) [23] and from experimentally determined transmittance parameters (Experimental Transmittance). Despite the uncertainty on the latter column’s $S_{0}$s sometimes being larger (all S polarization cases, and the initially P polarized with TGG **(III)**/**(I)** case) than the $S_{0}$ value, two significant figures for $S_{0}$ were still presented to allow for a comparison with the experimental and other simulated $S_{0}$ values.

Initial light polarization	TGG crystal	Position	Experimental $S_{0}$	Simulated $S_{0}$ (Reported Transmittance)	Simulated $S_{0}$ (Experimental Transmittance)
P (0°)	–	**(II)**/**(I)**	0.98±0.02	0.9950	0.97±0.01
P (0°)	No	**(III)**/**(I)**	0.500±0.008	0.4960	0.47±0.01
P (0°)	Yes	**(III)**/**(I)**	$6.5\times 10^{-4}\pm 0.1\times 10^{-4}$	$1.970\times 10^{-3}$	$3.3\times 10^{-4}\pm 0.01$
S (90°)	–	**(II)**/**(I)**	$3.45\times 10^{-4}\pm 0.06\times 10^{-4}$	$1.990\times 10^{-3}$	$3.4\times 10^{-4}\pm 0.01$
S (90°)	No	**(III)**/**(I)**	$1.29\times 10^{-4}\pm 0.03\times 10^{-4}$	$9.920\times 10^{-4}$	$1.7\times 10^{-4}\pm 0.006$
S (90°)	Yes	**(III)**/**(I)**	$2.7\times 10^{-5}\pm 0.2\times 10^{-5}$	$1.970\times 10^{-3}$	$3.4\times 10^{-4}\pm 0.01$

Second, there are three cases of significant difference between the experimental and simulated $S_{0}$ values, which are $S_{0}^{III}$ (**(III)**/**(I)**) for both polarizations without the TGG crystal for P polarized light and with and without the crystal for S polarized light. On the one hand, the experimental $S_{0}^{III}$s from an initial P polarization are larger than the simulated values; and on the other hand, the measured S polarization $S_{0}^{III}$s are much smaller than the simulation. Most of the discrepancies may be explained by scattered light from significant amounts of reflected light at each PBSC being measured in addition to the transmitted light. With a starting P polarization and the 45° Faraday rotation in effect from the TGG crystal, the majority of the light at the 45° PBSC is reflected resulting in a small energy recorded at **(III)** and consequently small $S_{0}^{III}$ (**(III)**/**(I)** ratio). However, the high intensity of reflected light could cause some of the reflected light at the 45° PBSC to scatter and be measured at **(III)**, resulting in a larger $S_{0}^{III}$ than expected. For S polarized light, smaller $S_{0}^{III}$s are observed with and without the TGG crystal. The discrepancy is larger when the TGG crystal is in place and could also be due to scattering contributions from the reflected light at PBSC 0 increasing the measured value of **(I)**. Additional losses in the TGG crystal or inaccuracies associated with measuring very small intensities could contribute to the discrepancies. The inaccuracy is a consideration, because the Gentec-eo QE25SP-S-MB has an effective minimum detectable energy of 4 μJ and the recorded mean energy at **(III)** for S polarized light with the TGG crystal was 8.3±0.6 μJ. The inaccuracy at such small energies is further supported in the large uncertainties relative to $S_{0}$ values on the simulated $S_{0}$s for initially S polarized case with experimental transmittance parameters shown in Table 1.

Third, given the agreement between the experimental and simulated results with the experimental transmittance parameters, a fit to the experimental data with the simulated Faraday isolator, Eq. (10), provided a check of the Faraday rotation. For the fit, the varying parameter was $\theta_{\mathrm{FR}}$ in the Faraday rotator Mueller matrix, Eq. (2), and the experimental data was necessarily the results including the TGG crystal, namely both initial polarizations for $S_{0}^{II}$ (**(III)**/**(I)**). The fit yielded an experimental Faraday rotation of $\theta_{\mathrm{FR}}=44\pm 1{}^{\circ}$.

The $S_{0}$ Stokes parameter analysis validates the construction and performance of the Faraday isolator. Utilizing the experimental transmittance parameters of $k_{1}=0.98\pm 2$ and $k_{2}=3.45\times 10^{-4}\pm 0.06\times 10^{-6}$ provides good agreement between experimental and simulated $S_{0}$ values, with observed discrepancies between the experiment and simulation explained by scattered light contributions. Furthermore, a fit to the experimental data yielded a Faraday rotation consistent with the expected 45° rotation.

### 7.2 Extinction ratio characterization

Imperfect transmissions and reflections in polarizers and Faraday rotators result in some amount of reverse propagating light to pass through a Faraday isolator; and this quality is often a measure of the Faraday isolator’s capabilities. Generally, the isolation ratio, extinction ratio, and isolation are similarly defined; but a variety of definitions exist [33], [34], [35], [36]. Here, the extinction ratio will be defined to match the experimental procedure, which used the same optical setup as the $S_{0}$ Stokes parameter comparison (Fig. 4). Thus, the extinction ratio presented here is

(12) $$ER=10\cdot \log_{10}\left(\frac{I_{0}}{I}\right)=10\cdot \log_{10}\left(\frac{\mathrm{(I)}}{\mathrm{(III)}}\right)=10\cdot \log_{10}\left(\frac{1}{S_{0}^{III}}\right)\;\left[\mathrm{dB}\right],$$

where ER [dB] is the extinction ratio, $I_{0}$ [W/cm^2^] is the laser intensity before entering the Faraday isolator, I [W/cm^2^] is the intensity after passing through the Faraday isolator in the reverse direction, **(I)** and **(III)** [mJ] are energy readings at the detector positions described previously in Section 7.1, and $S_{0}^{III}$ [unitless] is the $S_{0}$ Stokes parameter for position **(III)** also described in Section 7.1. The direct energy readings are interchangeable with the intensities here, because the laser beam diameter and repetition rate were constant.

**Fig. 6:**
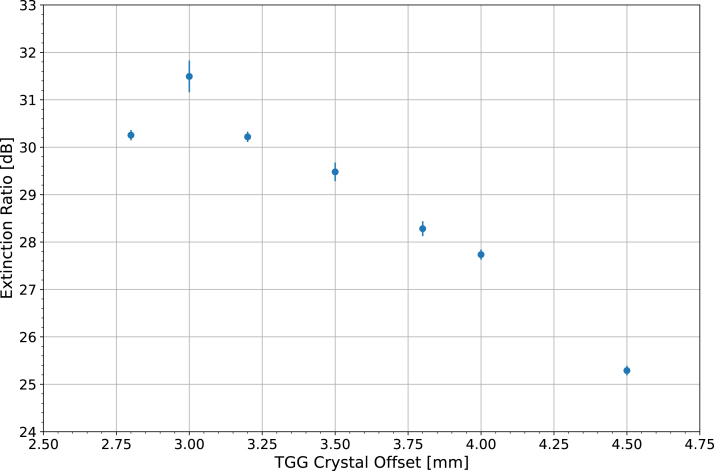
The extinction ratio (Eq. (12)) as a function of TGG crystal offset. The smallest possible offset distance was 2.8 mm, where the brass TGG Holder was flush with the magnets.

Measurements were recorded using the same method as previously described in Section 7.1 but only at positions **(I)** and **(III)**. HWP 1 was primarily set for P polarized light for easier measurement at **(III)**. Furthermore, the TGG offset position was varied to validate the optimum position. Fig. 6 displays the results of the tests for P polarized light. Fig. 6 demonstrates the most favorable TGG crystal offset is 3.0 mm, which has the largest magnitude extinction ratio of 31.5±0.3 dB for P polarized light. The polarization was also changed for the 3.0 mm offset, with HWP 1 rotated 45° and then another 45° (90° total from the initial HWP angle) corresponding to a linear polarization of S and then P again. By measuring the extinction ratio with of the two polarizations, the worst and best case back reflection scenarios are examined. Initially P polarized light is the worst case since the majority of light passes through the Vertical PBSC, yielding the largest $S_{0}^{II}$. Initially S polarized light is the best case, with the majority of light reflected at the Vertical PBSC, yielding the smallest $S_{0}^{II}$. Therefore, the extinction ratio for S polarized light is expected to be larger than for P polarized light, which is observed. The extinction ratio for the first 45° HWP 1 rotation corresponding to S polarized light was 39.9±0.2 dB and was 31.9±0.1 dB for the second 45° rotation back to initially P polarized light. Compared to the value in Fig. 6, the similar value of 31.9±0.1 dB measured with the return to P polarized light confirmed the reproducibility of the results.

### 7.3 Capabilities and comparison

The main characteristics of the Faraday isolator are:

• An extinction ratio at least 31.5±0.3 dB and as high as 39.9±0.2 dB.
• A clear open aperture diameter of 12 mm.
• An operating wavelength range of 1050 nm to 1080 nm.
• A laser damage threshold of at minimum 10 J/cm^2^.
• An upper laser power threshold around 700 W.

Importantly, the stated operating wavelength range may require adjustments in the insertion distance of the TGG crystal; however, the small change of the Verdet constant of TGG around 1064 nm [37] requires only small adjustments of the TGG crystal’s offset position. For example, a 2 Rad/(T m) increase in the Verdet constant corresponding to a 20 nm change in wavelength around 1064 nm [37] only requires an increase in the TGG crystal offset position by 1 mm. This operating wavelength range is first limited by the applicable bandwidth of the PBSCs [23] and second limited by the achievable Faraday rotation by the TGG crystal and magnets for a given wavelength. For the latter, the offset position limits the wavelength range without changing the dielectric medium or crystals. An upper limit on the offset position can cautiously be given as half the TGG crystal length, since the TGG still needs to be stable within the magnets; and a reasonably achievable lower limit on the offset position is 0 mm corresponding to the TGG crystal flush with the magnet edge. The required Verdet constant for 45° Faraday rotation can be calculated using the magnetic field models described in Section 5.3, which gives a wider scope of applicability. An offset of 20 mm corresponds to a Verdet constant of about 150 Rad/(T m), while an offset of 0 mm corresponds to about 37 Rad/(T m). With the Verdet constant of TGG as a function of wavelength [37], the wider operational range is between 600 nm and 1085 nm, assuming the PBSCs are adjusted to the correct wavelength. The mechanical system presented here can be further customized for different laser wavelengths by changing the PBSCs, dielectric material, or dielectric length as described in Section 5.3.

The components and the design of the Faraday isolator as a whole limit the extinction ratio. The limiting component is the TGG crystal based on the crystal size and anti-reflection coatings for the acceptable beam diameter and the laser damage threshold, respectively [22]. The $P_{max}$ value of TGG leads to the upper laser power threshold, where thermally induced depolarization is expected to have significant effects [18]. Compared to commercially available Faraday isolators, the damage threshold is comparable at values around 10 J/cm^2^ for 10 ns pulses [38], [39], given the limiting factor is the TGG crystal. Meanwhile, Faraday isolators and new dielectric materials reported in literature can have damage thresholds several orders of magnitude higher than commercially available ones [16], [17]. The under-performance compared to the state of the art isolators in this regard is expected, since the current Faraday isolator design was not intended for very high power lasers, where thermally induced depolarization is an issue.

The constructed Faraday isolator compares favorably to commercially available optical isolators, with respect to extinction ratios. Current high power optical isolators for 1064 nm light have quoted minimum extinction ratios from 30 to 38 dB and peak extinction ratios from 38 to 44 dB depending on the acceptable beam diameter [38], [39], [40]. The common trend across available isolators is a decrease in extinction ratio as the acceptable beam diameter increases, so commercial isolators with the same aperture as the presented isolator are a better comparison. The constructed Faraday isolator can utilize the full 12 mm diameter TGG crystal, and optical isolators with 12 mm diameter apertures have extinction ratios of at least 30 dB and up to about 35 dB [38]. Comparing the present isolator, the minimum extinction ratio corresponding to the initially P polarized results at 3.0 mm, which is 31.5±0.3 dB, is in line with commercial isolators. Additionally, the extinction ratio with initially S polarized light, which is 39.9±0.2 dB, is above the expected peak extinction ratio. Newly designed Faraday isolators for high power lasers are typically reported in the literature as having similar minimum extinction ratios around 30 dB [20]. Further, new dielectric materials or Faraday isolator configurations are typically calculated as obtaining similar minimum values, around 30 dB [15], [16], [17]. While one Faraday isolator achieved a high extinction ratio of 50 dB, this isolator was specifically designed for high extinction ratios [41]. Thus, the present isolator is also in line with other new Faraday isolators in terms of extinction ratio.

Finally, the total price of the constructed Faraday isolator, including the TGG crystal, 7 magnets, and two PBSCs, is around half the price of commercially available 12 mm acceptable beam diameter isolators. The Faraday isolator built from these designs and used for the validation experiments in Section 7 was first employed with a 50 Hz, 450 mJ, 6 ns pulse, 6 mm beam diameter, 1064 nm wavelength laser without issue. Moreover, the Faraday isolator has been used extensively with a 1 kHz, 60 mJ, 6 ns pulse, 3 mm beam diameter, 1064 nm wavelength laser. The total power through this second system has remained constant for over more than 2 years, with hundreds of operational hours. Thus, the two cases demonstrate the robustness and efficacy of the affordable Faraday isolator design presented here.

## CRediT authorship contribution statement

**Nicholas L. Wong:** Writing – review & editing, Writing – original draft, Visualization, Validation, Software, Methodology, Investigation, Formal analysis, Data curation. **Ben Delaney:** Validation, Software, Methodology, Data curation. **Takanori Miyazaki:** Validation, Software, Methodology, Investigation, Data curation. **Emma Sokell:** Writing – review & editing, Validation, Project administration, Methodology, Funding acquisition. **Fergal O’Reilly:** Writing – review & editing, Validation, Resources, Project administration, Methodology, Funding acquisition, Conceptualization.

## Declaration of competing interest

The authors declare that they have no known competing financial interests or personal relationships that could have appeared to influence the work reported in this paper.
